# Infrared Sensor Detection and Actuator Treatment Applied during Hemodialysis

**DOI:** 10.3390/s20092521

**Published:** 2020-04-29

**Authors:** Jian-Chiun Liou, Yu-Cheng Hsiao, Cheng-Fu Yang

**Affiliations:** 1 School of Biomedical Engineering, Taipei Medical University, Taipei 11031, Taiwan; 2 Graduate Institute of Biomedical Optomechatronics, Taipei Medical University, Taipei 11031, Taiwan; ychsiao@tmu.edu.tw; 3 Department of Chemical and materials Engineering, National University of Kaohsiung, Kaohsiung 811, Taiwan

**Keywords:** near-infrared light, sensor, far-infrared radiation, actuator, physiologically

## Abstract

Infrared thermography can be applied in different medical systems, for example it can be used to catch the images of living blood vessels. Far infrared rays can be used in a heating machine, which can be applied in the clinical hemodialysis patients. Infrared electronically sensitized images, which are generated by near-infrared Charge-coupled Device (CCD), are used to detect blood vessels, and used as a long-wavelength external stimulating therapeutic tissue repair system. When an infrared sensor detection and actuator treatment is applied during hemodialysis, a missing needle can be detected, and far infrared rays have a therapeutic effect on blood vessels. Because a far-infrared actuated light source can improve blood circulation, it is currently used to prevent fistula embolism in hemodialysis (HD) patients and reduce vascular occlusion after hemodialysis. Sensors used for sudden changes in heart rate variability (HRV) are used as predictive and evaluation indicators for our new method. Far-infrared actuated radiation can increase sympathetic nerve activity and regulation of parasympathetic and sympathetic nerves. We performed baseline measurements of the low-frequency/high-frequency ratio of autonomic nerve activity before hemodialysis (low frequency (LF), high frequency (HF), LF/HF, before HD) and after hemodialysis (LF/HF, after-HD). Based on data from the HRV continuity tracking report, 35 patients with autonomic nerve activation were treated and evaluated. We have demonstrated that the resulting near-infrared (NIR) sensor imaging and far-infrared actuator illumination can be used for the detection and treatment of hemodialysis patients.

## 1. Introduction

The novel and simple application case of infrared rays in a medical clinic is implemented by a hardware system that includes a large infrared array (1450 nm) with near-infrared (NIR) imaging chip array elements and far-infrared (1–20 μm) therapy for tissue repair. The infrared electromagnetic band is about 0.75 μm (750 nm)–1000 microns (μm). Depending on the energy content, the infrared band is divided into near infrared (near-IR, wavelength in the range of 0.75–2.5 μm), mid-infrared (mid-IR, wavelength in the range of 2.5–25 μm), and far infrared (far-IR, wavelength in the range of 25–1000 μm), respectively. The use of infrared wavelengths for different application technologies has basic theoretical limitations. The main applications of mid-infrared rays are detection, treatment, and analysis, and they can have a therapeutic function for patients. Because the mid-infrared has the detection function, it can be investigated as an infrared thermal imager (which is different from a near-infrared camera). Infrared rays of different wavelengths are useful and helpful for different detections, processes, and analyses. Regardless of the division method, instruments made with infrared as the working light source have been widely used in medical systems. Near-infrared rays can be applied for different medical applications, for example they can be used to capture different medical images, which are created by point-to-point human body temperature motion sensing detectors. Mid-infrared can be used in infrared gas analyzers and infrared thermal imagers, and far infrared rays can be used in therapeutic heating of clinical patients.

Intravenous infusion is an important method for clinical treatments. Selecting a superficial vein in the back of the hand, inserting a needle, and performing an infusion are some of the most common clinical tasks for clinic medical staffs. Whether the procedure is done for ordinary patients’ treatments, emergency interventions, venipuncture, rapid establishment of venous access, supplementation with IV fluids, or administration of a blood transfusion, completing it as soon as possible is particularly important to prevent or alleviate patients’ discomfort and suffering. Traditionally, medical staffs use veins on the hands; to expand the blood vessels, they apply a rubber tube to bind the veins, use simple visual judgment, and then inject the medicine or fluid. Consistent effective performance relies primarily on experience, which can cause clinical staff to have great mental stress. The subcutaneous structure of human tissue is complex, and in some cases, it is difficult for staff to puncture a vein using only their clinical skills and experience.

There is a huge variety of veins, including fragile veins, vaginal veins, highly mobile veins, superficial small veins, and pediatric cephalic veins. Some are not easy to see, so it is difficult to properly puncture them, and the process is highly prone to clinical error, potentially causing the patient fear and pain. The development of an assistive angiographic apparatus could resolve such problems, improve the efficiency of medical work, and reduce other related risks. Currently, blood vessel imaging devices on the market incorporate a light-source module, a camera module, an image-processing module, and a projection module [1,2,3,4,5,6]. The image that indicates vein direction depends on the camera angle, and some thinner blood vessels cannot be photographed, so the application has limitations. At present, the image projection angle can only be adjusted manually by experienced operators. 

Equipment disassembly and reassembly can introduce error in the projection angle, which in turn increases the error rate for placing the venous needle. In addition, a single light source is used, and the resulting imaging effect is poor. Present-day equipment is large and inconvenient for medical personnel to access when required. Biological tissue optical imaging employs optical radiation and can be used for clinical diagnoses and detection. Interactions between light and biological tissues are complex. Differences in the wave characteristics of light in biological structures, and in the physical and chemical properties of light can significantly alter the resulting images. Different biological tissues can require different light sources to achieve usable imaging. Light in the range of red to near-infrared (NIR) is said to be in the diagnostic and treatment window [7,8,9,10]. When light is irradiated onto a biological tissue, the most important occurrence of light in the visible-NIR band is in the form of scattering. At this time, the light quickly diffuses into the irradiated materials during the propagation process, which is more conducive to contact with and reflective absorption by tissues. The differences in light wavelengths caused by the scattering effect are small. The scattering effect can occur anywhere in most tissues, from cell membranes to organelles. Nuclei and mitochondria in organelles are the most important causes of scattering. In biological tissues, light absorption and scattering can cause attenuation of the light, which can be expressed by the effective light attenuation coefficient (*μeff*) [11,12]:

*µeff*=(0+√(3*μa*(*μa*+*μ*^’*s*)))/1

where *μ’s* =*μs*(1−*g*) is the light propagation scattering coefficient, and it is the anisotropy of the tissue, with a typical value of 0.9.

The attenuation of light in tissues and the combined effect of absorption can be detected with the naked eye. Studies have shown that although fat is one of the larger components of tissues, its ability to absorb light is weak. For the red to NIR region, hemoglobin in the blood has strong absorptive properties. Oxygenated hemoglobin (HbO2) and deoxygenated hemoglobin (deoxyhemoglobin, Hb) have different spectral properties. There are different chromophores in the tissues, including water, fat, oxygenated hemoglobin, deoxyhemoglobin, and melanin. The main component of the skin is water, and the melanin content of the epidermis is almost negligible, while subcutaneous fat accounts for about 10%–40% of skin, so melanin concentration is higher there.

Venous blood vessels up to 8 mm deep can be made visible, making these relatively easy targets for delivering injections and blood transfusions. Infrared is sensitive to thermal sensing and reveals the location of blood vessels. When the arm or any other part of the body is irradiated, the machine takes an infrared image that reveals the blood vessels, because infrared rays can penetrate skin and tissues better than visible light. It is known that 95% of the dry weight of red blood cells in the blood is hemoglobin, and hemoglobin in venous blood is deoxyhemoglobin. Taking into account differences in skin colors between individuals, we selected light sources with wavelengths of 1225 ± 10 nm and 1300 ± 10 nm, so that the veins would be clearly discernible in different individuals. The main reason is that the test results can be supplied to applications in different fields. The principle of infrared to obtain the fingers’ images is used for “body recognition (finger vein recognition)”. NIR imaging can recognize the colors of the skin on palms. As the fist is clenched for a duration of 30 s, the palm’s skin color will change into white. Once the fist is released, if the palm returns to normal skin color within 3 s, the result suggests that the blood vessels of the heart have good elasticity and they do not have the problem of serious blockage. If the fingertips’ color quickly turn back to red, it means that the blood pressure is normal. If it takes more than 5 s to restore the original skin color, it means that the elasticity of the blood vessels may not be good, so we need to be careful if there is an arterial occlusion or arteriosclerosis. The principle of infrared to obtain the images of the veins in the back of the hand is used to “inject blood vessels”.

## 2. Infrared Treatment

A special feature of far-infrared rays when they are applied to the human body is their penetrating power and consequent strong warming effect; the temperature of the skin and subcutaneous tissues increases. Physiologically, the human skin and subcutaneous tissues undergo a warming effect, and the whole body is warmed evenly and comfortably. At the same time, far-infrared rays resonate with and activate human cells. Humans are very water-rich organisms. In adults, water accounts for 60% of our body weight, 40% of which is in cells as intracellular fluid and 20% is in plasma (16%) and interstitial (4%) fluids. Thus, when far-infrared rays act on cells, they mainly cause vibrations of the water molecules inside and outside cells, and they convert aged macromolecules into smaller molecules by activating water molecules, proteins, and cells. Local action can dilate microvessels, accelerate blood flow, open side sacs, ameliorate microcirculatory disorders, improve the blood supply and oxygen supply to cellular tissues, accelerate the absorption and dissipation of inflammatory exudates, strengthen metabolism, and improve the body’s overall health status.

Microvascular dilation can promote blood circulation, working against congestion and other disorders that impede metabolism, as well as clearing and restoring tissues and promoting enzyme growth. Old waste and harmful substances retained in the body are discharged along with the metabolized contents of sweat glands, and cosmetic residues present in the pores can be directly discharged from the skin and sweat without passing through the kidneys, thereby avoiding an increased burden on the kidneys. These benefits can be confirmed by the results of low-temperature far-infrared radiation at a temperature of about 40 °C. As we know, infrared rays can be absorbed by any substance and cause a thermal reaction. As far-infrared rays are applied for deep penetration, wavelengths in the range of 8–14 µm are the same as those of human body radiation. According to a large number of authoritative clinical reports, far-infrared rays of the same wavelength have good physiotherapeutic effects on the human body [13,14,15,16,17,18]. 

Far-infrared rays can penetrate deeply into human skin and subcutaneous tissues and promote blood circulation, helping the body maintain a steady temperature. Rays generated by far-infrared heating elements in this band are used for physiotherapy. The spectrum also possesses electromagnetic wave energy that can be quickly absorbed by the body. Far-infrared spectral energy can cause atoms and molecules in the body to vibrate, generating heat and resulting reactions that elevate the deep skin temperature.

Hypertension and arteriosclerosis are caused by the autonomic nervous system, endocrine regulatory system, small arterial vasoconstriction, and narrow blood vessels [19,20,21,22,23,24]. The use of far-infrared radiation technology to expand microvessels under the skin can promote blood circulation in blood vessels to reduce high blood pressure and can also improve symptoms of hypotension. To ameliorate joint pain, far-infrared can penetrate deep into muscles and joints, warm the body from the inside, relax muscles, drive oxygen and nutrient exchange via the microvascular network, and eliminate the accumulated fatigue substances kept in the body, and waste such as lactic acid to eliminate swelling. Soreness can often be effectively relieved.

## 3. Design Architecture and Simulation

The wavelength range of high-order near-infrared (NIR) is approximately 0.76–1.60 μm, and this is used for imaging technologies in smart medical care systems. It is applied to hemodialysis arteriovenous fistulas in vascular anastomosis procedures. Infrared illuminates the blood vessels and reveals 8 mm veins. As noted above, when far-infrared radiation is directed at the human body, the temperature of the skin and subcutaneous tissues increases. The novelty of this paper is that we structured a two-serial connection perovskite CuIn_x_Ga_(1−x)_Se_2_ (CIGS) material for detecting blood vessels and providing tissue repair therapy. The ratio of element concentrations was adjusted to complete the bonding structure between the four elements. In addition, the combined structure had tandem CIGS films, meaning the top perovskite CIGS film was able to absorb most of the visible light and the bottom CIGS composite material was able to absorb near-infrared and far-infrared radiation. The perovskite CIGS materials were serially connected, making the conversion of CIGS films more efficient. Physiologically, the entire body was warmed evenly and comfortably.

**Figure 1:**
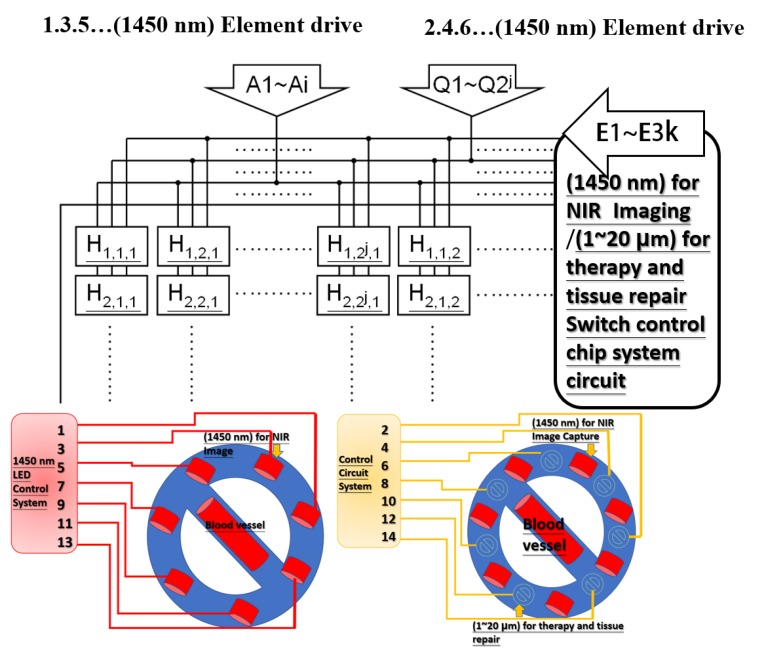
Switching control system circuit of 1450 nm for near-infrared (NIR) imaging (1–20 μm), which can be used for therapy and tissue repair.

This research designed and implemented a hardware system that included an infrared emitter of 1450 nm with a large array as NIR imaging chip array elements and far-infrared of 1–20 µm for therapy and tissue repair. For using an infrared beam of 1450 nm with a large array as NIR imaging, the switching control wafer system circuit contains chip array elements, and an infrared beam of 1–20 µm for treatment, therapy, and tissue repair occurs simultaneously with imaging and illumination of the skin and tissues. The output power of the designed system is adjustable based on the severity of the patient’s wound. Figure 1 shows the switching control system circuit for NIR imaging (with wavelength of 1450 nm) and for therapy and tissue repair (with wavelength of 1–20 μm). As Figure 1 shows, H_i, j, k_ represents the three-dimensional signal controller, which corresponds to the LED array element. A1–Ai generate the array drive signals of devices with 1450 nm (for NIR imaging) and Q1–Q2j generate the array drive signals of devices with 1–20 μm (for therapy and tissue repair). The “k” switching signals generate the enabling signals and let the system have the functional operation (as the switching control).

**Figure 2:**
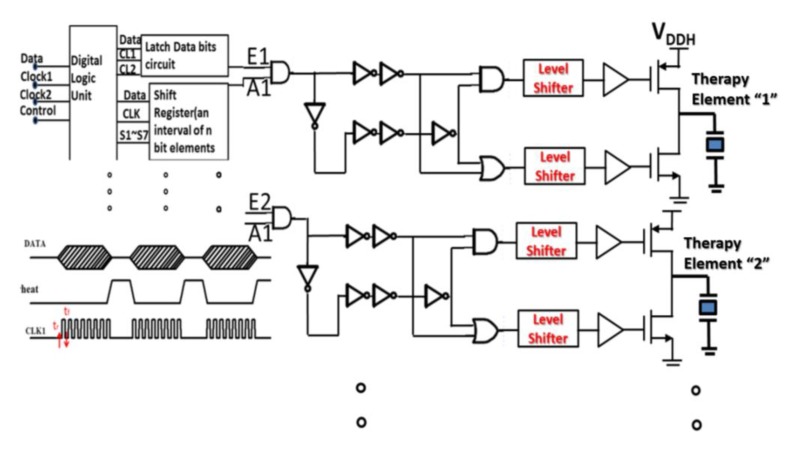
Far-infrared logic operation control system.

Infrared at 1450 nm is used for NIR image chip array elements, and infrared at 1–20 µm is used for therapy and tissue repair. The advantages of this system are fast imaging speed and high resolution. To avoid the large array far-infrared system becoming overheated, after the logic operation of the circuit design, the output signal is supplied to the large array infrared of 1450 nm for NIR imaging chip array elements and the infrared of 1–20 µm for treatment through a high-voltage driving circuit. To avoid overheating the large arrays of the far-infrared systems during tissue repair, sequential operation of spacer components is required, as shown in Figure 2.

These logic gates have a digital operation function, which can be applied to avoid the excessive current density of the entire system at the same time. Far-infrared logic operation control system is designed to avoid turning on the high-power far-infrared array elements at the same time. These signals with the same of “A1” are used to drive high-power components, but they are skillfully designed to be turned on at different times. The designed “enable E1” is turned on corresponding to the time of “Therapy Element 1”, and the “enable E2” is turned on corresponding to the time of “Therapy Element 2”. These designs can save power consumption and avoid the damage problem of excessive current density.

## 4. Experiment and Results

**Figure 3:**
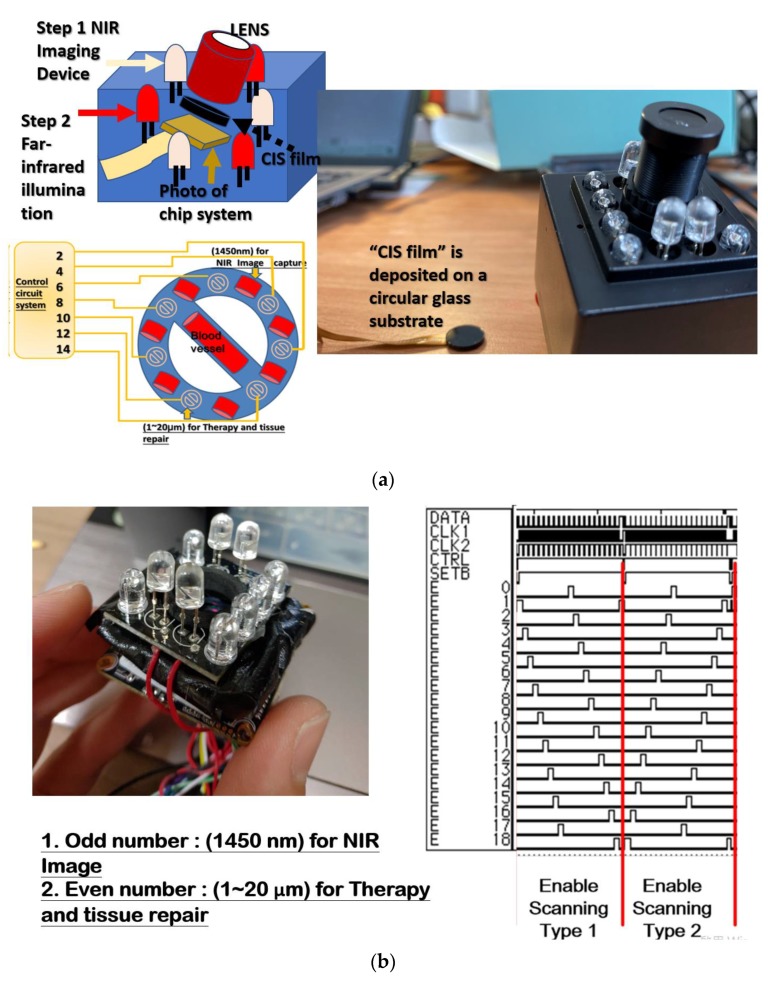
(**a**) The image of the CuIn_x_Se_2_ (CIS ) system. (**b**) in the large far-infrared array, odd and even channel groups are turned on at different times to avoid them interfering with each other.

CuIn_x_Se_2_ (CIS) film is deposited on a circular glass substrate; after that the device with CIS film is integrated and packaged into the “photo of chip system”, then the “photo of chip system” with CIS device is combined with the LENS, as shown in Figure 3a. The system combines far infrared radiation and near infrared for vein detection. The first step of this system is to capture the image of the floor tube position with near-infrared images and perform hemodialysis with a needle. The second step is to irradiate the kidney fistula with far infrared rays, which can effectively prevent the fistula from hardening and prolong the life of the fistula. This system uses both near-infrared radiation for vein detection and far-infrared radiation for treatment.

This research implemented a hardware system that includes a large infrared array (1450 nm) for NIR imaging chip array elements, and infrared (1–20 µm) for therapy and tissue repair, as shown in Figure 3b. From the input data signal (few count signal), the actual signal is verified by the operating system. This controlled system is regarded as a signal driven by being optional to prevent the positioning of adjacent elements in the space by delaying the touched position signal design time (ΔT); the delay time (ΔT) is enough time for the finger to point to another position. The signals are processed by multiple flip-flops to prevent signals at the same location from overlapping. The large array element decoding/encoding system integrated a handheld miniaturized far-infrared array image system, by which the methods for far-infrared images were designed and implemented.

### 4.1. Light Source

The light source is based on the principle of a semiconductor light-emitting element, and it has very good luminescent properties. The precise light wavelength distribution is within the range of NIR light. Infrared sensors are sensitive to heat, and a person’s blood vessels are warm, so the position of the blood vessels can be seen. In infrared light, blood vessels under the skin are readily visible. Many medical units use this technology when they draw blood, but deficiencies remain. The flow of blood vessels can be seen at once, but drawing blood is still based on touch. Light near 1225 nm is used as the main light source, and light near 1300 nm is used as the auxiliary source, depending to some extent on differences in skin pigmentation between individuals. Illuminating the skin with light sources of 1225 ± 10 nm and 1300 ± 10 nm allows one to directly observe the vein with the naked eye, eliminating unnecessary error. More direct observation of finer veins can better assist medical staff in performing venipuncture to improve the auxiliary function of the instrument.

For biometric vein recognition (palm vein recognition), the venous blood vessel identification system uses very safe bio-vascular signature recognition technology. Images of the fingers, palm, and dorsal veins of the hand are acquired by the principle of using hemoglobin in the blood to absorb infrared light. Using an infrared Charge-coupled Device (CCD) camera, the veins are easily distinguished. The characteristic image is extracted according to a dedicated alignment algorithm. Digital images of the venous blood vessels are first stored in a computer data storage system. The vein recognition machine immediately makes a vein map, extracts the feature value, and then uses a specific noise filtering. Special image distribution binarization and fine processing techniques are used to distribute the digital information from the image. To extract features, information is commonly stored in the processor host for venous eigenvalue comparisons to authenticate individuals and confirm their identity.

In regard to the concept and technical principle of vein recognition, today’s vein recognition technology has four characteristics: high anti-counterfeiting capacity, ease of use, rapid identification, and high accuracy. The probability of two people having the same vein structure is 3.4 billion to 1, so it can be said that each person’s vein structure is virtually unique. More importantly, vein recognition must be live-body recognition, meaning it can only be performed using the finger of a live individual. Vein recognition uses hemoglobin in the blood to absorb infrared rays. When NIR light is applied to the finger or palm, the hemoglobin in the subcutaneous vein of the finger or palm is relatively close to the infrared illumination in comparison with other physiological tissues. The absorption rate is high, so the black-and-white contrast of the image is clear. Under illumination with NIR light, muscle tissue is light and blood vessels are dark, making the vascular structure readily detectable.

**Figure 4:**
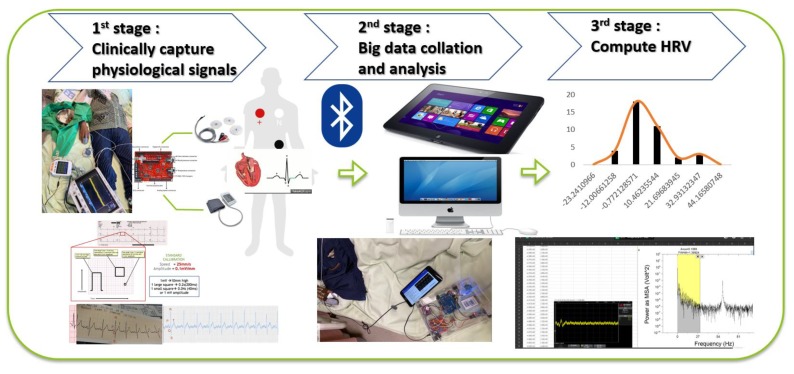
The block diagram of complete methodology.

Unlike traditional fingerprint recognition requirements with respect to finger humidity and fingerprint integrity, vein recognition is not affected by whether the finger is wet, clean, or damaged, because the skin is not scanned, only the veins under the skin. Finger recognition was chosen because NIR light easily penetrates the skin to image the vein structure. The principle of the palm vein technique is based on the fact that hemoglobin in the blood of the palm absorbs infrared light. The image-capturing system for the palm is small and sensitive to infrared wavelengths. The image to be stereoscopically photographed is the shadow of the vein. The next step is to perform a feature recognition comparison of the blood vessel pattern. The system is micro-processed to produce a blood vessel pattern feature distribution image. The processing system used high-level algorithms with complex operations to check a database and confirm the individual’s identity. The block diagram of complete methodology is shown in Figure 4; heart rate variability HRV is obtained from the external device and the “pre processing” steps are divided into three stages. The first stage is to obtain the physiological signals, which are clinically captured by the external heart rate acquisition system, as shown in Figure 4. The entire signal acquisition process is used to explain to the patients the process of monitoring physiological signals, and then to capture the patients’ heart rate signals. The second stage is the collation and analysis of big data, which analyzes and collects the heart rate variability data after hemodialysis (HD). The third stage is “Compute HRV”, in which patients’ heart rate cycle signals are converted into frequency distribution by Fourier transform. The HD-performed patients have different complications; undergoing hemodialysis while under observation for the extent to which heart rate variability (HRV) affects their sympathetic and parasympathetic nerves.

**Figure 5:**
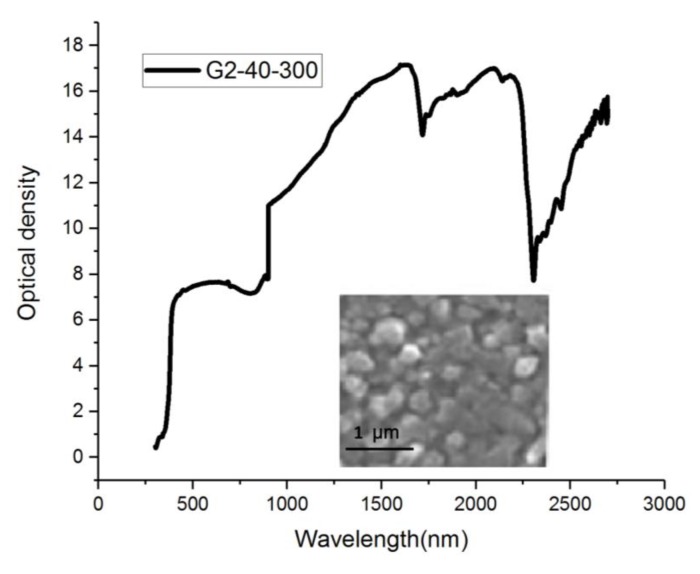
Proportions of individual elements of different CuIn_x_Ga_(1-x)_Se_2_ (CIGS) films in the wavelength distribution of the optical spectrum (G2-40-300).

**Figure 6:**
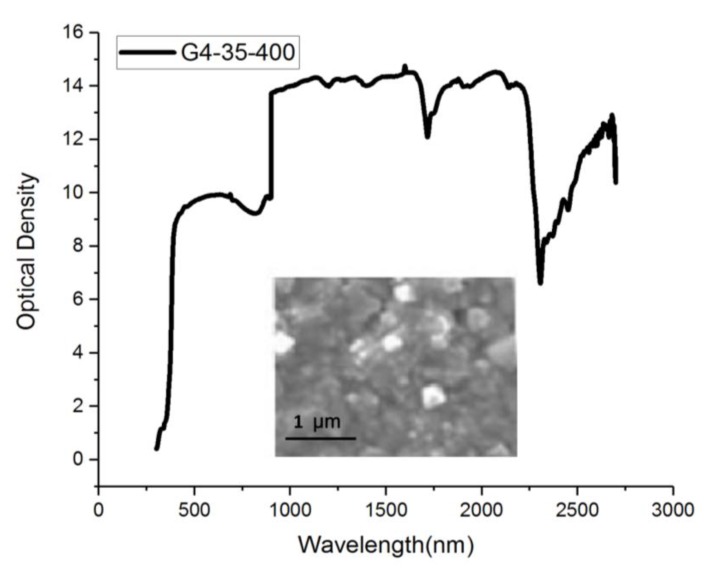
Proportions of individual elements of different CIGS films in the wavelength distribution of the optical spectrum (G4-35-400).

When *x* = 1, CuIn_x_Ga_(1−x)_Se_2_ will form a synthetic CIS material. Figure 5 shows the CIGS composite material that results when 2 Ge elements are added to the synthetic CIS material. The deposition time is 40 min at 300 °C, and the wavelength after light wave filtration is between 1000 and 2300 nm. The intensity is 17 units, and it can be used for image retrieval. Figure 6 shows the CIGS composite material. The deposition time is 40 min at 300 °C, and the wavelength after light wave filtration is between 1000 and 2300 nm. The intensity is close to 14 units, and it can be used for image capture.

**Figure 7:**
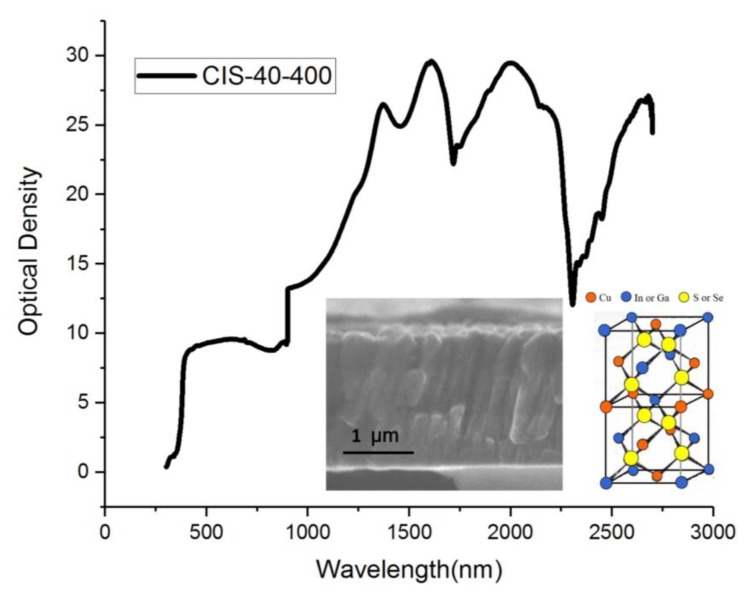
A filter with a bandpass wavelength of 1000–2300 nm was placed in front of each imaging system to eliminate ambient light.

The deposition time of the CIS material is 40 min at 400 °C, and the wavelength after light filtering is between 1000 and 2300 nm. When the intensity is normalized, it is close to 30 units, and it can be used in the treatment system. Far-infrared rays of the same wavelength have good physiotherapeutic effects on the human body. The filter wavelength of the CIS film was complete at this stage. The measured spectrum is shown in Figure 7. A filter with a bandpass wavelength of 1000–2300 nm was placed in front of each camera to eliminate ambient light. NIR converted the measurement data in this wavelength range into an adjustable process parameter, which is important proportional operation information. This experimental procedure is important as it can enhance filter materials to optimize parameter definitions or modify parameters. Such infrared element function experiments can easily be used to check irregular surfaces. NIR is not destructive for them, and little or no sample preparation is required. It can also be used to analyze multiple components in one scan. The ratio of the proportion of individual elements of different CIGS films in the wavelength distribution of the optical spectrum is shown in Figure 5 and Figure 6.

**Figure 8:**
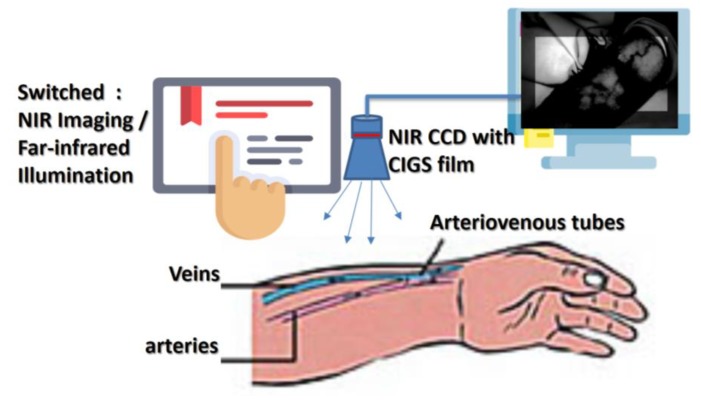
Diagram of infrared hemodialysis architecture.

Figure 8 is a schematic diagram for determining from the NIR image how long high-power infrared therapy is needed. Renal patients receiving long-term dialysis must undergo surgery. Renal patients use their own blood vessels (autologous arteriovenous ducts) or artificial materials (artificial blood vessels) to connect arteries and veins to create vascular access. Long-term hemodialysis can be achieved by switching between infrared imaging and far-infrared irradiation treatment. In this way, enough blood flow can be provided to supply hemodialysis sufficient to sustain life.

**Figure 9:**
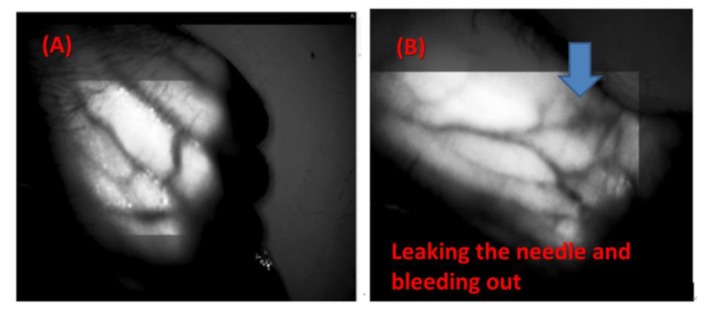

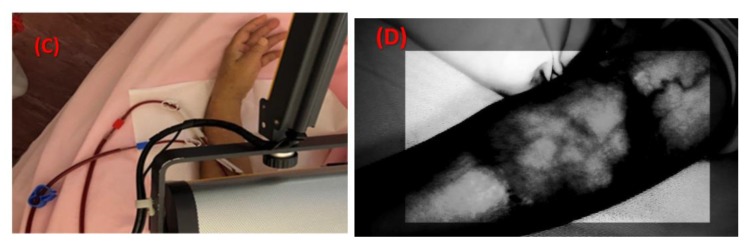
Observing arteriovenous vascular images and therapeutic tissue repair: (**A**) near-infrared source, very clear image of blood vessels, which can be observed in a local range, (**B**) leaking needle and bleeding out, (**C**) in the hemodialysis process, the near-infrared imaging system can be used to simultaneously observe needle placement, needle leak, and blood seepage, and (**D**) during the hemodialysis process, with NIR image of a puncture in the affected area.

Compared with other NIR imaging systems for venous detection, the innovation of this new technology is to add special materials that increase the absorption wavelength (1000–2300 nm) and thereby allow the dynamic observation of arteriovenous blood vessel images and therapeutic tissue repair, as Figure 8 shows. Figure 9A shows the use of the near-infrared source on blood vessels, generating a clean image that can be observed in a local range. Figure 9B shows a leaking needle (extravasation) and bleeding out. Figure 9C shows that as in the hemodialysis process, the near-infrared image system can be used to simultaneously observe needle placement, whether there is a needle leak, and blood seepage. Figure 9D shows the NIR image during hemodialysis.

### 4.2. Far-Infrared Implementation

1. Promoting the entry of water into the blood vessels in tissues to enable dialysis to proceed smoothly.

The far-infrared system for each treatment (40 min, a distance of 20–25 cm, room temperature of 22–25 °C, and body temperature of 36–37 °C, which can be increased by 0.1–0.2 °C. At the same time, with expansion of the tube, the pressure inside the tube drops, so the water in the tissue can quickly enter the blood vessel, causing the edema to disappear and keeping the blood pressure relatively stable.

2. Understanding the elasticity and size of blood vessels.

The temperature of the skin is affected by changes in ambient temperature and body temperature, and subcutaneous blood vessels expand or contract in response. Using the far-infrared radiation therapy system to maintain the skin temperature at 40 °C, along with sufficient water, one can observe the limit of the blood vessels’ expansion. One can also see the increase in blood flow in an arteriovenous fistula, indicating whether it is hardened or still elastic and requires further treatments. Because many patients have neuropathic problems, the impact on the surrounding tissues is slow, and it is a good way to determine the degree of vasodilation. This method can be used for differential diagnoses when blood flow due to an arteriovenous fistula is reduced.

3. Help with artificial blood vessels.

When a wound does not bleed, it takes 40 min to treat the wound once a day, which can accelerate wound healing, reduce the deformation of artificial blood vessels, and reduce the chance of bacterial infection. At any time and place, including the palm of the hand, the back of the hand, the sole of the foot, and the back of the foot, treatment can increase blood flow to the entire hand or foot by 20%–30%. This is a simple and effective method to avoid artificial embolization.

4. Direct energy supply.

The limbs of an arteriovenous fistula (three times the normal blood flow) can quickly absorb the resonance energy of far-infrared rays. After treatment, attention should be paid to the warmth of the limb, because the limb can also relatively easily dissipate heat and cause the body temperature to drop. The best time for treatment is during gastrointestinal discomfort or malnutrition. Uremia patients have poor physical fitness. Directly supplying heat energy using a far-infrared treatment system is a direct and rapid method. Clinicians should pay attention to the warmth of the ambient environment to ensure a proper therapeutic effect.

5. The effect on arteriovenous fistulas after surgery.

If there is no bleeding after an operation, this treatment can be used the same day under a doctor’s supervision. Starting the following day, the patients can self-illuminate the surgical site on a daily basis, so blood flow can be quickly increased to facilitate recovery. It is better to do the treatment with the individual in a supine position. Because the venous intima can quickly thicken, it is possible to avoid venous obstruction by continuously aligning using a far-infrared treatment system and compressing the vein for 1–3 mos after surgery, so that the rate of vein enlargement is greater than the rate of intimal hyperplasia. If there is slight local infection, it is better to supplement treatment with antibiotics, but the ointment should not be applied first. Vein compression is a method of using blood pressure to accelerate expansion of the vein. Each time, pressure should be applied for about 6 s; the more times, the better, and if the blood flow each min is greater than the flow required for dialysis, then the parameter can be changed occasionally.

6. Phlegm and pain relief.

After dialysis or vasodilation, if the limb has symptoms such as blood stasis, swelling, and pain, on the second day the affected limb can be raised higher than the heart, and treatment with the far-infrared ray therapy device can immediately reduce swelling and pain. If a patient has an aneurysm, then a physician must be consulted. A thrombosis is prone to occur in varicose veins, and far-infrared therapy is the best alternative to oral anticoagulants.

**Figure 10:**
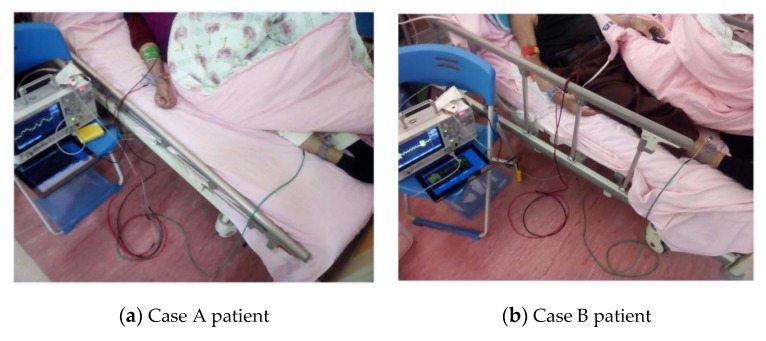
Hemodialysis while observing the extent of heart rate variability affecting sympathetic and parasympathetic nerves.

Figure 10 shows images of case A and case B patients, who have different complications, undergoing hemodialysis while under observation for the extent to which heart rate variability (HRV) affects their sympathetic and parasympathetic nerves.

**Figure 11:**
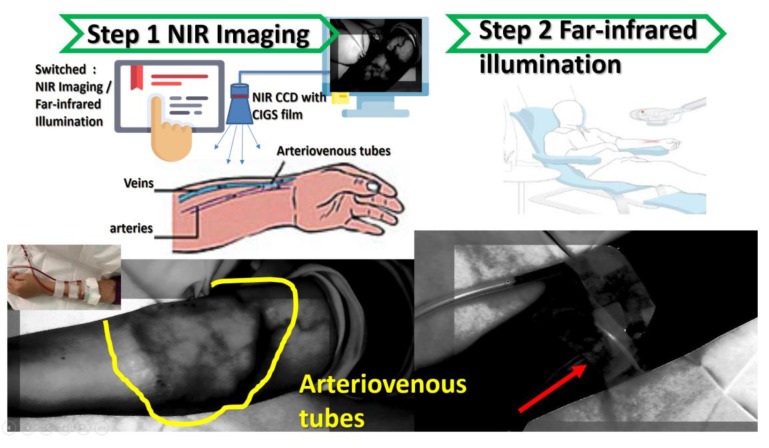
The diagram of both NIR for veins detection and far infrared for therapeutic effect.

The diagram of both NIR for veins detection and far infrared for therapeutic effect is shown in Figure 11, this system combines far infrared radiation and near infrared for vein detection. The first step performed by the investigated system is to capture the near-infrared images of the floor tube positions and then to perform hemodialysis with a needle. The second step is to irradiate the kidney fistula with far infrared rays, which can effectively prevent the fistula from hardening and prolong the life of the fistula. The system uses both NIR for vein detection and far-infrared radiation for treatment. Infrared irradiation on the dialysis fistula is used to promote local blood circulation, improve the blood flow of the fistula, and prevent the formation of embolization in the renal dialysis fistula. This irradiation process can reduce the number of times kidney dialysis patients need to perform fistula surgery due to a fistula embolization; thereby the kidney dialysis patients can have a more stable quality of life and relieve the physical discomfort caused by the kidney dialysis process. The biggest difference from the existing vein detection method is that the existing vein detection method only exists individually and independently for infrared imaging. This study has combined the functional mechanism of far-infrared treatment, so that the status of the venous sphenoid tube can be observed at the same time, and the treatment can prevent fistula sclerosis and prolong the life of the fistula.

Table 1 lists the main clinical features. We studied 35 emergency hemodialysis patients with continuous noninvasive monitoring of severe blood pressure, heart rate increase, or risk of morbidity within 4 h of admission. All severe trauma patients that could be monitored were studied and were not excluded. Non-invasive hemodynamic monitoring was started during hemodialysis for 4 h (average 240 ± 10 min), and patients were followed up to the treatment room for emergency instruction. In addition, the database also contains the following data: age, gender, poor nutritional status, poor hypertension control, aortic aneurysm, and with far-infrared treatment. The proportion of cases for the item (number of item cases/total *n*) is shown in Table 1. The proposed symptom observation and computer program is an information system, which is directly connected to the patients. It can use the difference between after hemodialysis and before hemodialysis. The monitoring system provides real-time data and online display of calculations to inform patients without affecting the patients’ treatments or ability to provide medical care.

**Figure 12:**
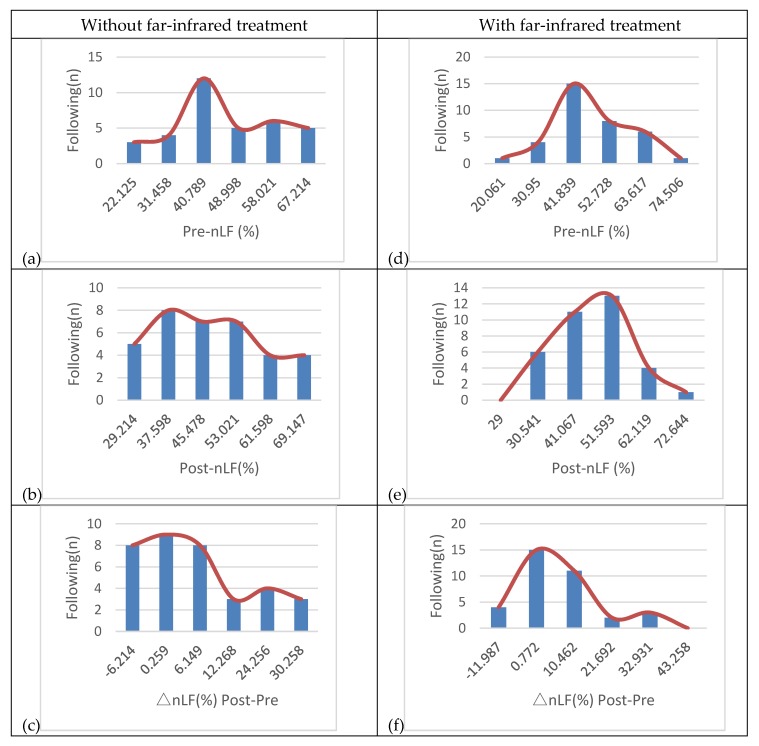
Without far-infrared treatment: (**a**) LF/HF before-HD, (**b**) LF/HF after-HD, (**c**) autonomic nerve activation observed; with far-infrared treatment (**d**) LF/HF before-HD, (**e**) LF/HF after-HD, and (**f**) autonomic nerve activation observed. Note: hemodialysis (HD)

Figure 12 shows images of 35 patients who have different complications and in the cure treatment of hemodialysis, while under observation for the extent to which heart rate variability (HRV) affects their sympathetic and parasympathetic nerves. Figure 12a–c show that 35 patients had LF/HF before-HD, LF/HF after-HD, and autonomic nerve activation observed without infrared treatment, respectively; Figure 12d–f show that 35 patients had LF/HF before-HD, LF/HF after-HD, and autonomic nerve activation observed with infrared treatment, respectively. The different results for without and with far-infrared treatments prove the “treatment and analysis” data, and the results shown in Figure 12 belong to a very concentrated normal distribution.

HRV is an autonomic nervous system total activity index, HF is a parasympathetic index, and LF is a sympathetic and parasympathetic activity indicator. The percentage of high-frequency components (HF%, equal to HF/(HF+LF)) is a parasympathetic activity index, and the percentage of low-frequency components (LF%, equal to LF/(HF+LF)) is a sympathetic activity index. LF/HF is a sympathetic/parasympathetic balance indicator, where LF and HF are in the frequency ranges of 0.04–0.15 Hz and 0.15–0.4 Hz. Using HRV continuity tracking reports, 35 patients with autonomic nerve activation were observed, and the results are shown in Figure 12. Among them, 15 patients with autonomic nervous disorders were well regulated. The difference between LF/HF before-HD and LF/HF after-HD was only 0.772. When the 15 patients had autonomic dysfunction, the difference was about 10–12. However, when 5 patients had autonomic nervous disorders that were poorly regulated, the difference was about 21–32. The HRV continuity tracking report indicates the autonomic neurological disorders of the 5 patients were of the first type, with high sympathetic activity and a decrease in parasympathetic activity.

## 5. Conclusions

This study demonstrated that an infrared electron sensitization image could be investigated as a system for blood vessel detection and therapeutic tissue repair. Establishing vascular access for observation and treatment in a central vascular clinic is the first step for treating patients with renal failure, and protecting blood vessels is another care priority. Because the heartbeat is at any given time affected by the sympathetic and parasympathetic nervous systems, this effect will be reflected in the degree of change in heart rate. For this reason, heart rhythm variation is used as an indicator of therapeutic effect. The investigated system is a non-invasive, simple way to assess autonomic function. Hence, it is suitable for measurement and evaluation in the general population and has become a standard physiological indicator. The investigated system can also be used for HRV analysis to study the autonomic function of hemodialyzers. Our results showed that the infrared irradiation effect before hemodialysis and after hemodialysis was associated with a decrease in HRV and autonomic dysfunction.

## Figures and Tables

**Table 1:** List of the main clinical features.

	Patients (*n* = 35)
Age, mean ± SD	68 ± 12
Gender, *n* (%)	
Male	18/35
Female	17/35
Problem:	
Poorly controlled hypertension	32/35
Aortic aneurysm	16/35
Detour	28/35
With Far-Infrared treatment	35/35
